# Drowsiness Detection Using Ocular Indices from EEG Signal

**DOI:** 10.3390/s22134764

**Published:** 2022-06-24

**Authors:** Sreeza Tarafder, Nasreen Badruddin, Norashikin Yahya, Arbi Haza Nasution

**Affiliations:** 1 Department of Electrical and Electronic Engineering, Institute of Health and Analytics, Universiti Teknologi PETRONAS, Seri Iskandar 32610, Malaysia; sreeza_19001742@utp.edu.my (S.T.); norashikin_yahya@utp.edu.my (N.Y.); 2 Department of Informatics Engineering, Faculty of Engineering, Universitas Islam Riau, Tembilahan 28284, Indonesia; arbi@eng.uir.ac.id

**Keywords:** drowsiness detection, electroencephalography, ocular artifacts, machine learning, ensemble learning

## Abstract

Drowsiness is one of the main causes of road accidents and endangers the lives of road users. Recently, there has been considerable interest in utilizing features extracted from electroencephalography (EEG) signals to detect driver drowsiness. However, in most of the work performed in this area, the eyeblink or ocular artifacts present in EEG signals are considered noise and are removed during the preprocessing stage. In this study, we examined the possibility of extracting features from the EEG ocular artifacts themselves to perform classification between alert and drowsy states. In this study, we used the BLINKER algorithm to extract 25 blink-related features from a public dataset comprising raw EEG signals collected from 12 participants. Different machine learning classification models, including the decision tree, the support vector machine (SVM), the K-nearest neighbor (KNN) method, and the bagged and boosted tree models, were trained based on the seven selected features. These models were further optimized to improve their performance. We were able to show that features from EEG ocular artifacts are able to classify drowsy and alert states, with the optimized ensemble-boosted trees yielding the highest accuracy of 91.10% among all classic machine learning models.

## 1. Introduction

Drowsy driving has become a worldwide concern because it causes numerous fatalities on roads annually. According to the National Safety Council, drowsy driving causes approximately 100,000 accidents, 71,000 injuries, and 1550 deaths annually [1]. According to the American Automobile Association, drowsy driving accounts for 9.5% of all accidents [2]. Drowsiness refers to the moment immediately before sleep onset. During this time, a person feels sleepy and finds it difficult to keep their eyes open. Drivers traveling long distances often drive in a drowsy state. Other factors that can lead to drowsiness are sleep deprivation, monotonous driving, and alcohol consumption. The effects of drowsiness on driving include loss of focus, slow reaction times, and poor judgment, which can be detrimental to drivers and other road users. Drowsiness-related motor vehicle accidents are likely to result in serious injuries and death, which can have a considerable socioeconomic impact. Hence, efforts should be made to prevent such accidents, including the development of systems to detect driver drowsiness. There have been numerous investigations for an effective and accurate driver drowsiness detection system over the past decades. Primarily, these methods can be categorized into two types based on the source of the data or measurement: vehicle- and driver-based [3]. Table 1 summarizes the different methods used to detect drowsiness.

Vehicle-based methods use measurements obtained from vehicles, such as lane deviations, braking patterns, and steering wheel grip, as indicators of driver drowsiness [4,5,6]. Compared to driver-based methods, vehicle-based data require less processing and are more straightforward to interpret. However, individual driving skills, habits, and environmental factors such as weather and road conditions may lead to inaccurate results. Driver-based methods can be further divided into those that capture the driver’s behavior using cameras placed in the vehicle and those that measure the driver’s physiological signals using sensors attached to the body. Some of the investigated visual behavioral indicators include drooping posture, blink frequency, yawning, head movement, head position, gaze direction, blink duration, fixed gaze, frequent nodding, and sluggish facial expressions [7,8,9,10,11,12,13]. Visual-based methods have the advantage of being non-intrusive; however, the detection accuracy can be affected by poor lighting conditions, differences in image angle, poor image resolution, and occlusions such as eyeglasses. However, non-visual techniques that use physiological signals are not affected by these factors and can be a viable alternative for detecting signs of drowsiness. Furthermore, physiological signals can provide a more accurate measure of drowsiness owing to their strong relationship with driver fatigue. Some of the physiological signals that have been used are electroencephalogram (EEG), electrocardiogram (ECG), electrooculogram (EOG), photoplethysmogram (PPG), galvanic skin response (GSR), and functional near-infrared spectroscopy (FNIRS) [14,15,16,17,18,19,20]. EEG signals are used to analyze brain states such as sleep, alertness, fatigue, and stress and are most commonly used in sleep-related research [21]. Various time and frequency features of EEG signals have been used for drowsiness detection in previous studies. The main issue in using EEG in real-world driving conditions is the wearability of the EEG devices. Efforts have been made to address this issue by reducing the number of electrodes required; however, this may lead to reduced accuracy. To overcome this, other studies have proposed hybrid approaches in which an EEG is complemented by an ECG [22] or an EOG [23]; however, this requires extra ECG or EOG sensors be placed on the body.

In this study, we adopted a different approach to finding a complementary indicator of drowsiness from EEG signals. It is well known that an EEG signal captures not only neural activities but also artifacts, which are undesirable electrical signals from other physiological activities and movements. The most prominent type of artifact is the ocular artifact, which is caused by eyeblinks and eye movements. In most EEG analyses, the conventional approach regards these artifacts as contaminants and removes them during the preprocessing stage by using any of the available eyeblink artifact removal techniques. However, we contend that because an EOG has been used in combination with an EEG for drowsiness detection and that many of the visual-based techniques in driver drowsiness detection rely on features extracted from the eyes [10,24], there is a possibility that similar information can also be extracted from the eyeblink artifacts in an EEG. One possible application of our work is a system that combines conventional EEG analyses for drowsiness detection, such as power spectrum analysis and connectivity with ocular information, which are also extracted from the same EEG signal. The advantage of this system over the hybrid EEG–EOG drowsiness detection system is that it does not require the placement of additional sensors around the eye area.

To the best of our knowledge, few studies have been conducted to determine whether drowsiness can be detected from ocular features extracted from EEG signals that have not undergone artifact removal [25,26]. Therefore, in this study, we attempt to answer the question: “Can we use ocular indices extracted from EEG signals contaminated with eyeblink artifacts as drowsiness indicators”? In this investigation, we used BLINKER software developed by the authors in [27] to extract ocular indices from EEG signals. The embedded feature selection method was then used to select the most effective and valuable ocular parameters obtained from BLINKER for the classification. In this study, we investigated both traditional machine learning techniques and deep learning methods for classification.

The main contributions of this study are twofold. First, this study explores the potential of using features or measurements extracted from ocular artifacts in EEG signals for the detection of driver drowsiness. In doing so, a novel method of drowsiness detection can be developed, which is based on features extracted from EEG signals that have not undergone the ocular artifact removal process. This new method can complement existing drowsiness detection based on frequency domain analysis of EEG signals. Second, the features and classification methods that achieved the best classification performance were investigated.

The remainder of this paper is organized as follows. A literature review of related studies is presented in Section 2, followed by a detailed description of the methodology in Section 3. Section 4 presents the results and discussion, and Section 5 concludes the paper.

## 2. Related Work

Numerous studies have been conducted to successfully detect drowsiness among drivers, and blink-related parameters have been widely investigated. Previous research has shown that blink duration and frequency can be used to indicate driver drowsiness [28,29]. However, most of this type of research uses image-based techniques to extract the blink parameters for drowsiness detection. Researchers have used different types of cameras to collect eye images or video recordings of a driver’s face, which are then processed to extract features related to their eyes [29,30,31]. Vision-based drowsiness detection has some limitations. It can be affected by problems such as different lighting conditions, the eye region being outside the image frame, and occlusions such as spectacles or sunglasses. According to [32], EEG-based drowsiness detection is better than the visual method because the drivers have to wear masks due to COVID-19. The preprocessing of the EEG signal includes linear filtering and wavelet threshold denoising.

Of all the non-visual techniques, EEG-based methods are the most predictive and reliable for drowsiness detection. However, very few studies have explored the use of ocular artifacts in EEG signals to extract blink-related features such as blink duration, blink frequency, and blink amplitude to detect driver drowsiness. A helmet-based physiological signal monitoring system that differs between alert and drowsy states by detecting blinking and heart rate variability (HRV) is discussed in [33]. The results showed that blinking duration (higher than 400 ms) and eye-opening time increased during the sleepiness state compared with the alert state. Blink signals were collected from the raw data and processed to obtain six different features: blink duration, closing time, reopening time, positive peak, negative peak, and interval. The drowsiness detection technique presented by Kartsch et al. [34] is based on behavioral and physiological studies of subjects using EEG signals. The blink duration was calculated using a single channel, and an alarm was triggered when the average blink duration exceeded a given threshold of 500 ms. The detection accuracy of the system was 85%. The researchers in [10] used a combination of EEG and EOG signals to measure blink duration for driver drowsiness detection.

Some of the most up-to-date EEG-based techniques include feature extraction and classification as part of the drowsiness detection process [35]. Feature extraction typically involves the extraction of different frequency bands from EEG signals using techniques such as discrete wavelet transform (DWT), fast Fourier transform (FFT), independent component analysis (ICA), principal component analysis (PCA), and autoregression (AR). The classification methods used include support vector machine (SVM), K-nearest Neighbors (KNN), naïve Bayes classification, decision trees, ensemble methods, artificial neural network (ANN), linear discriminant analysis (LDA), etc. FFT was used to extract features from EEG data in [36,37,38] but used different classifiers. A KNN classifier with k = 3 yielded the best accuracy of 95.24% in [30], an SVM yielded the best accuracy of 83.71% in [37], an ANN yielded a better accuracy of 86.5% compared with an SVM in [32], and linear regression was found to provide an accuracy of 90% in [39]. In [40], the KNN classifier obtained an accuracy of 91% when applied to features extracted using short-time Fourier transform (STFT) and was found to outperform an LDA and an SVM when performing classification from features extracted using time analysis [40]. However, an SVM outperformed a KNN when using the determinism (DET) feature extracted by the recurrence quantification analysis. Priya et al. [41] used a publicly available EEG dataset where the subjects’ eye state was mentioned and trained a KNN model to predict drowsiness. The study focuses on different feature engineering techniques to boost the accuracy of the KNN model up to 98%. An ANN was used in [42] to train chaotic features and the logarithm of the EEG signal energy and in [43] on a combination of EEG features that were selected using an LDA. Therefore, it can be concluded that the best classifier or machine learning technique depends heavily on the features that have been extracted to perform the classification. This implies that any investigation of new features for drowsiness detection must include various machine learning techniques to achieve the best results.

Based on the literature review, blink-related parameters, such as blink duration, eye-opening time, and eye-closing time, were measured using image-based drowsiness detection techniques. However, EEG-based drowsiness detection techniques use spectral analysis. Most EEG-based drowsiness detection methods use spectral analysis and the extraction of different frequency bands in the EEG signal. The work closest to our proposed technique is [34], where the blink duration was extracted from the EEG signal and used as the first-level indicator of drowsiness. However, our work conducts further investigation by considering all other measurements that can be extracted from the eyeblink artifact of an EEG, not just the blink duration. The literature has shown that different machine learning and deep learning techniques have been successfully used to perform classification from EEG signals. Therefore, to obtain the best performance in classifying drowsy and alert states from ocular features in EEG signals, it is necessary to investigate a few types of classifiers.

## 3. Methodology

**Figure 1:**
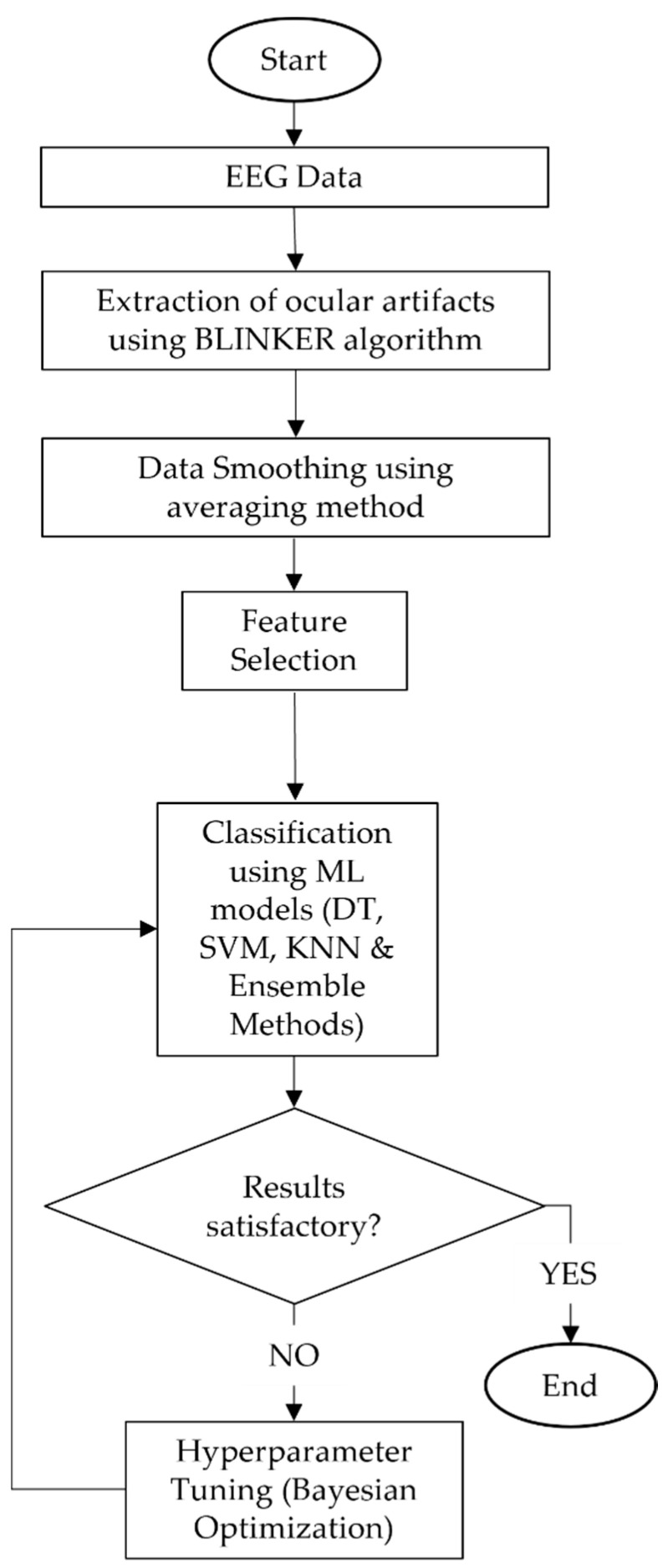
Flowchart of the research methodology.

This section discusses the methodology used in the study. Figure 1 shows a flowchart of the methodology. Details of the methodology are elaborated in the subsequent subsections.

### 3.1. Dataset

The public dataset collected in [44] was used in this study. EEG signals were recorded from 12 healthy participants by using a neuroscan amplifier with 40 channels in a simulator-based driving environment. The EEG recordings were performed in two phases. The first phase lasted for 20 min, while the second phase was continuous driving for 40 to 100 min duration until the subject reported driving fatigue. The EEG in the last 5 min of the first phase was marked as a normal or alert state, and the last 5 min of the second phase was marked as fatigue or drowsy state. The sampling frequency used was 1000 Hz.

**Figure 2:**
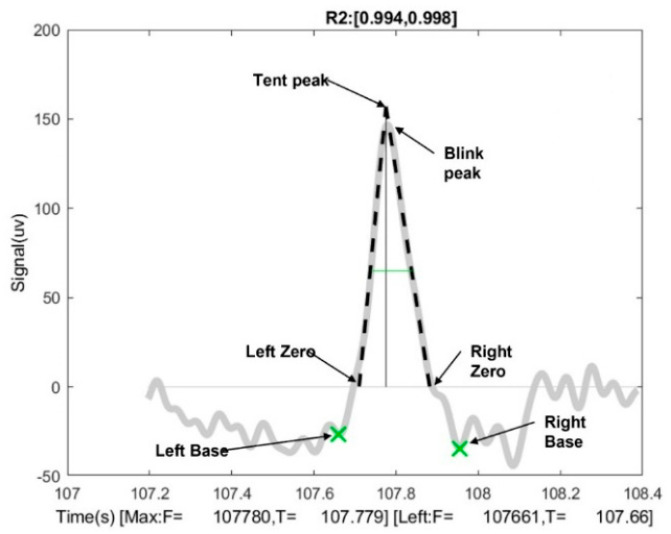
A schematic diagram of an eye-blink signal with the various blink landmarks used by the BLINKER software [27].

The BLINKER toolbox [27], which works on the MATLAB platform, was used to extract ocular indices from EEG eyeblink artifacts. BLINKER detects the intervals and potential blinks created from the EEG signal when the signal is more significant than 1.5 standard deviations above the overall signal mean. It considers only those possible blinks that stretch for more than 500 ms and are at least 50 ms apart. BLINKER employs a tent-fitting method to define blinks because typical blinks have rounded, tent-like shapes. Figure 2 shows a schematic of a sample blink artifact with various blink landmarks. LeftZero is the last zero crossing. If the signal does not cross zero between the current blink and the previous blink, LeftZero is the frame with the lowest amplitude. A similar definition is applied to the RightZero frame.

The interval between LeftZero and maxFrame is the upstroke, whereas that between maxFrame and rightZero is the downstroke. For each potential blink in a candidate signal, BLINKER computes the best linear fit for the inner 80 percent of the upstroke and downstroke as represented by dotted black lines in Figure 2. The first local minimum to the left of the maximum velocity frame during the upstroke is LeftBase. In the downstroke, RightBase is the first local minimum to the right of the maximum-velocity frame. TentPeak is the intersection of the upstroke and downstroke. In a stereotypical blink, TentPeak is slightly ahead of and above the maximum of the actual blink trajectory, which is called BlinkPeak.

The proximity of a potential blink to a typical blink is measured by its quality, which is denoted by R2 in this example. Simple assessments of how closely the blink matches a stereotypical blink are provided by the values of R2 and the relative position of TentPeak to BlinkPeak. This specification removes many tiny quick eye movements without eliminating genuine blinks. BLINKER chooses the best signal to characterize the shapes and properties of the blinks. It provides 25 blink-related parameters for a set of potential blinks. Table 2 lists the descriptions of these parameters.

### 3.2. Preprocessing

In the preprocessing stage, eyeblink artifacts were first extracted from the EEG signals corresponding to the alert and drowsy states of each subject using the BLINKER algorithm and labeled accordingly. Table 3 summarizes the number of blink occurrences extracted from each subject for alert and drowsy states.

Data cleaning was performed to remove missing values, corrupted values, and incomplete features. Finally, data smoothing was performed by averaging five consecutive data points to reduce random variations in the raw data.

### 3.3. Data for Training, Validating, and Testing

After preprocessing, 1963 eyeblink artifacts with 25 features were selected for training and testing. This resulted in 49,075 features that were sufficient for training and testing the models. Of the 1963 eye-blink artifacts, 1288 eyeblink artifacts belonged to the “Drowsy” class while the remaining 675 eyeblink artifacts belonged to the “Alert” class. For predictive modeling, it is important to split the data into training, validation, and testing sets, which allows the development of a highly accurate model. The training set was the dataset used to train the model, and the model learned the underlying patterns from the training set. The validation set was used to validate the performance of the model during training, and the test set was a separate set of data used to test the model after training. In this study, 70% of the data was used to train and validate the models, and the remaining 30% was used to test the models.

A subject-independent k-fold cross-validation technique was applied to train the models. In this study, 10-fold cross-validation was used to prevent overfitting. This approach randomly divides the dataset into 10 groups/folds, and each fold is approximately the same size. For each iteration, 1 group was considered as a hold-out or test dataset and the remaining 9 groups were considered as the training set. In this way, 10 iterations were performed so that each data point was trained. In each iteration, the method returned an accuracy score, and the average of the accuracy scores was used as the consolidated cross-validation accuracy score.

### 3.4. Investigation of Classic Machine Learning Models and Ensemble Methods

#### 3.4.1. Feature Selection

There are three types of feature-selection methods: filter, wrapper, and embedded. In this study, the embedded feature selection technique was applied to obtain the best features out of 25 features provided by BLINKER. This method performs better than other methods because the feature selection process is performed while training the model, and the most valuable features are selected to achieve better performance [45]. The embedded method comprises decision trees that represent a feature-based process in which each decision tree is formed by extracting random features. A subset of the dataset was created, and different combinations of features were tested to obtain the best accuracy. The predictive model was then trained based on the best accuracy provided by a subset of features.

#### 3.4.2. Choice of Classifiers

This study focuses on the binary classification problem in which the classes are fatigue/drowsy and alert. The classifiers used in our investigation were decision tree (DT), K-nearest neighbor (KNN), and support vector machine (SVM).

A decision tree (DT) is a supervised machine-learning algorithm in which the root node is used to decide based on specific parameters. The branches from the node correspond to the possible outcomes of the nodes and are connected to the next decision node. Leaf nodes are the final outcomes that typically represent class distributions or labels [46]. In this study, a fine tree and an ensemble of decision tree classifiers were trained. Gini’s diversity index was used as the split criterion, and a split of 100 was used to train the model. On the other hand, the optimized DT model has been hyperparameter-tuned using the maximum number of splits ranging from 1 to 1374 and 2 different split criteria, Gini’s diversity index and maximum deviance reduction. Because small trees make decisions more quickly than large trees do, they are much easier to see and understand.

This principle behind the ensemble models for machine learning is to combine multiple models to improve the overall performance. Among various ensemble techniques, bagging and boosting are the most popular and were used in this study [47]. The bagging method is used to reduce variance in high variance classifiers, such as decision trees. Several subsets of data from the training dataset are chosen using row sampling with replacement and fed to the base learners in parallel [48]. The base learners (DT) were trained on a subset of the data and provided an output. The outputs from all DT models were aggregated, and the final output was obtained based on majority voting. Unlike bagging, the basic principle behind boosting is to apply homogenous machine learning techniques sequentially, with each ML method attempting to enhance the model’s stability by focusing on the errors produced by the previous ML algorithm [49]. The main difference among all variants of the boosting approach is how each base learner’s mistakes are regarded as improved by the following DT in the sequence. We applied AdaBoost, one of the most widely used boosting algorithms, which assigns equal weights to each sample of the training dataset when training the first weak DT. The subsequent weak learner model is trained using the recalculated weights of the sample to present a misclassification from the previous model. The AdaBoost algorithm performs prediction by recalculating the weighted average of the weak models.

The K-nearest neighbor (KNN) algorithm predicts the similarity between the seen and unseen data points. The KNN algorithm is much faster than algorithms that require training, such as the support vector machine (SVM), because it only stores training data and does not learn from it. In this study, the traditional KNN model was trained using K = 3. The model was further optimized using Bayesian optimization in which the number of neighbors, the distance metric, and the distance weight were considered.

The support vector machine (SVM) is a popular supervised learning algorithm used for regression and classification. The primary approach of the SVM is to find the decision boundary/hyperplane that maximizes the distance between the data points of different classes in an N-dimensional space, where N represents the number of features. Data points closer to the decision boundaries are called support vectors, which influence the maximization of the hyperplane [50]. In this study, we used a fine Gaussian SVM with a kernel scale of sqrt (P)/4, where P is the number of predictors and a hyperparameter-tuned SVM, where the kernel function, kernel scale, and box constraint level were tuned before model training to improve the model performance and prediction.

#### 3.4.3. Hyperparameter Tuning

Hyperparameter optimization in machine learning aims to find the hyperparameters of a given machine learning algorithm that return the best performance as measured on a validation set [14]. Finding the best combination of hyperparameters can be difficult, but it is possible to automate the process using different optimization methods such as grid search, random search, and Bayesian optimization. Bayesian optimization was used in this study. With this approach, the algorithm tracks past evaluation results used to form a probabilistic representation of the model performance. This was performed using an objective function as the primary evaluator of the hyperparameter. Bayesian optimization aims to reduce errors by using more data. This approach continuously updates the surrogate probability model after each evaluation of the objective function [51]. For the decision tree model, the hyperparameters chosen for tuning were the number of splits and the split criteria. The distance metric, the distance weight, and the number of neighbors (K-value) were selected for tuning in the KNN classifier, whereas for the SVM model, the box constraint level, kernel function, and kernel scale were the hyperparameters that were tuned. Finally, for the ensemble models, the hyperparameters selected for tuning were the ensemble method, number of learners, learning rate, maximum number of splits, and the number of predictors to be sampled.

### 3.5. Performances Metrics

The performance of each model was analyzed using the following performance metrics.

#### 3.5.1. Sensitivity/Recall/True Positive Rate (TPR)

The true positive rate (TPR) measures the predictive model to correctly identify the true positives (TP). TPR is the ratio of correctly predicted actual positives to the total number of observations in the actual class, which includes false negatives (FN). A higher TPR value indicates that the predictive model classifies the actual positives more accurately. The TPR is given by (1).

(1) $$\mathrm{TPR}=\frac{\mathrm{TP}}{\mathrm{TP}+\mathrm{FN}}$$

#### 3.5.2. Fallout/False Positive Rate (FPR)

The false-positive rate (FPR) is the total number of false positives (FPs), which are negative observations incorrectly classified as positive observations, divided by the total number of negative observations. For any predictive model, the FPR value should be low, indicating that the predictive model accurately classifies true negative (TN) observations. The FPR is given as

(2) $$\mathrm{FPR}=\frac{\mathrm{FP}}{\mathrm{FP}+\mathrm{TN}}$$

#### 3.5.3. Miss Rate/False Negative Rate (FNR)

The total number of positive observations incorrectly classified as negative observations divided by the total number of positive observations is the false-negative rate (FNR) or miss rate. The FNR value should be low, as it indicates that the model classifies true positives more accurately and is given as

(3) $$\mathrm{FNR}=\frac{\mathrm{FN}}{\mathrm{FN}+\mathrm{TP}}$$

#### 3.5.4. Precision

Precision or positive predictive value (PPV) is the ratio of true positives to the sum of true and false positives. The precision value ranges from 0 to 1, and higher values indicate that the predictive model performs better in classifying true and false classes. The precision is given as

(4) $$\mathrm{Precision}=\frac{\mathrm{TP}}{\mathrm{TP}+\mathrm{FP}}$$

#### 3.5.5. Accuracy

The accuracy is the ratio of correctly classified observations to the total number of observations. This is a significant measure for evaluating predictive models. It summarizes performance based on the number of correct predictions (TP and TN). However, they do not provide much information on false positives and false negatives. Accuracy is calculated using (5).

(5) $$\mathrm{Accuracy}=\frac{\mathrm{TP}+\mathrm{TN}}{\mathrm{TP}+\mathrm{FP}+\mathrm{FN}+\mathrm{TN}}$$

#### 3.5.6. F1-Score

The F1-Score is an excellent evaluation metric for determining the balance between precision and recall and when the class distribution is uneven. The F1-Score is given as

(6) $$F1-\mathrm{Score}=2*\frac{\left(\mathrm{Precision}*\mathrm{Recall}\right)}{\left(\mathrm{Precision}+\mathrm{Recall}\right)}$$

#### 3.5.7. ROC–AUC

The receiver operating characteristic (ROC) provides a summary of the performance of a classifier. The TPR is plotted along the y-axis; the FPR is plotted along the y-axis, and the threshold value used for the observations of a particular class can be adjusted. The area under the curve (AUC) indicates the accuracy of the model in terms of separability. A predictive model is better than random guessing if the AUC value is greater than 0.5. A model is considered good if its AUC value is larger than 0.8.

## 4. Results and Discussion

**Figure 3:**
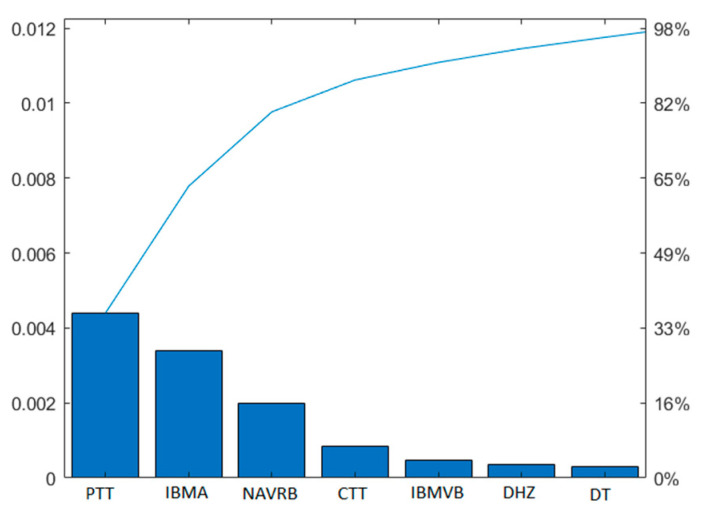
Selected features using the embedded feature selection technique where the line graph above the bar shows the cumulative sum of the importance values.

The tree-based embedded method selected 7 useful features out of the 25 features provided by BLINKER, which increased the accuracy of the predictive models. The features are the peak time tent (PTT), inter-band maximum amplitude (IBMA), negative amplitude velocity ratio base (NAVRB), closing time tent (CTT), inter-link maximum velocity base (IBMVB), duration half zero (DHZ), and duration tent (DT). Figure 3 shows the best features selected from the 25 features. The y-axis on the left and right shows the importance value of each bar and the cumulative percentage of the feature importance, respectively. The x-axis represents the feature indices. The line graph above the bar shows the cumulative sum of the importance values. Feature importance indicates the variables that are more relevant to the target classes. The feature importance value of 98% shows that the seven selected features can improve the predictive model’s performance and reduce the computation cost. These seven features were used to train the decision tree, KNN, SVM, ensemble of bagged trees, and ensemble of boosted tree classifiers.

Fine DT, fine KNN, fine Gaussian SVM, and an ensemble of bagged and boosted tree classifiers were trained and further hyperparameter-tuned to observe the differences in their performance. The accuracies and other performance metrics are listed in Table 4.

If we look at the other performance metrics in Table 4, even though the fine DT and the optimized DT are 82% successful in classifying the data points into alert and drowsy observed from the AUC value, the hyperparameter-tuned DT performs slightly better than the fine tree in observing the FPR and FNR. The model was hyperparameter-tuned using the maximum number of splits criterion, Gini’s diversity index, and maximum deviance reduction. Bayesian optimization yielded an accuracy of 80.40% when the maximum number of splits was 211 and the split criterion was Gini’s diversity index. After hyperparameter tuning, the accuracy improved by just 0.2%. However, when we investigated the confusion matrix and the ROC–AUC curve, this model might perform poorly, even though it provided over 80% accuracy. When the FPR and FNR are higher for any model, the model misclassifies the true class as false and vice versa. A decision tree’s aim is to reduce the training data into the smallest possible tree, and this is done by separating the nodes into numerous sub-nodes and repeating the procedure during model training until only homogenous nodes remain. For the fine DT, the number of splits used was 100, and for the hyperparameter-tuned model, the number of splits was 211. Because small trees make decisions more quickly than large trees and are much easier to understand, this might be a reason why the hyperparameter-tuned DT does not show much improvement in terms of performance.

For the fine KNN, the number of neighbors used was K = 3 along with the Euclidean distance metric. For the hyperparameter-tuned KNN model, the hyperparameters selected were the number of neighbors (between 1 and 688), the distance metric (city block, Chebyshev, correlation, cosine, Euclidean, Hamming, Jaccard, Mahalanobis, Minkowski, and Spearman), and the distance weight (equal, inverse, and squared inverse). The best model performance was obtained when the number of neighbors was three, the distance metric was Mahalanobis, and the distance weight was the squared inverse. From the evaluation metrics of fine KNN and optimized KNN, it is clear that the optimized KNN has better separability (AUC of 0.93) than the fine KNN model (AUC of 0.90) as shown in Table 4. In both KNN models, before and after hyperparameter tuning, the optimal K value was three. However, after hyperparameter tuning, the separability and accuracy of the model increased by 0.3% and 2.9%, respectively. The selection of different distance metrics may result in a better performance in the hyperparameter-tuned model.

A traditional fine Gaussian SVM was trained with the Gaussian kernel function; the kernel scale was 0.66, and the accuracy was 82.50% as shown in Table 4. The optimized SVM model was trained using different box constraint levels ranging from 0.001 to 1000, kernel scale values ranging from 0.001 to 1000, and Gaussian, linear, cubic, and quadratic kernel functions. The Bayesian optimizer optimized all the hyperparameters and yielded the highest accuracy (86.20%) when the kernel function was cubic, the kernel scale was 1, and the box constraint level was 28.9228. Even though both models before and after hyperparameter tuning had similar separability (AUC = 0.91), the FPR and FNR values were quite different. For example, the FNR of the Gaussian SVM was 16.9%, whereas after tuning, it was found that a cubic SVM yielded an FNR of 12.5%. This indicates that hyperparameter tuning reduces the number of false negatives when a cubic kernel function is used. This also led to a higher accuracy than the Gaussian SVM.

Ensembles of bagged and boosted tree classifiers were also trained and hyperparameter-tuned using Bayesian optimization for better predictive performance. The accuracy of the bagged tree classifier was 85.10% and that of the boosted tree classifier using AdaBoost was 79.10%. To train the bagged tree classifier, several subsets of data from the training dataset were selected using row sampling with replacement and fed to the base learners in parallel. The outputs from all the base learning models were aggregated, and the final output was obtained based on majority voting. Although, in general, the boosted tree classifier is better because it tries to solve the errors of the base learners sequentially and builds up the model, it did not show promising results on our dataset as boosting can be sensitive to outliers. Both models were further optimized by tuning the hyperparameters. The hyperparameters selected for tuning were the number of learners (50–500), learning rate (0.001–1), the maximum number of splits (1–1374), and number-of-predictors-to-sample (1–8).

**Figure 4:**
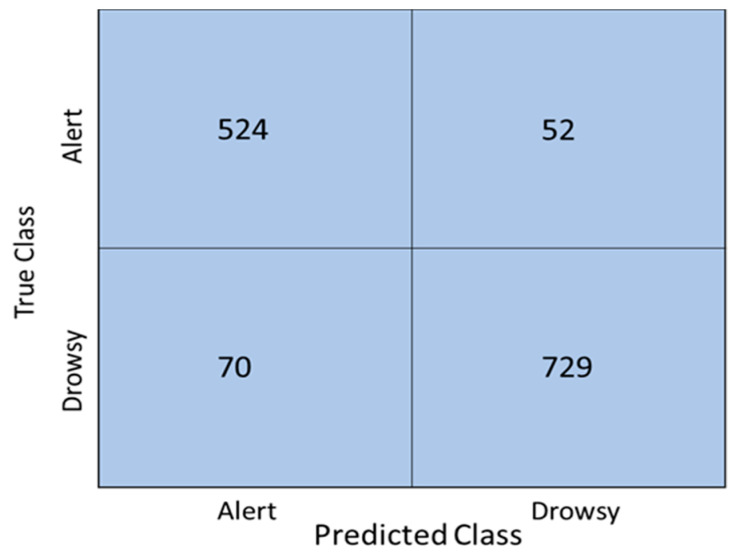
Confusion matrix of the optimized ensemble AdaBoost method.

With hyperparameter tuning, the best performance of the bagged tree classifier was obtained using 498 learners and 302 splits. However, the improvement in accuracy after hyperparameter tuning was not significant, with an increase of 1.3% to 86.4. For the boosted tree classifier, the best performance was obtained using AdaBoost, with a maximum number of splits of 84 and 100 learners. The accuracy improved to 91.1%, and this value was the highest achieved among all the models used in this study. The number of misclassified data points was also small compared to the other ML models studied as shown in the confusion matrix in Figure 4. The AUC value is 0.97, indicating that there is a 97% chance that this model can correctly separate both classes. The F1-Score was 0.90, which indicates a better balance in the precision and recall of the model.

Accuracy is an excellent performance metric when there is an equal number of observations in both classes. In addition to accuracy, precision/TNR, fallout/FPR, recall/TPR, miss-rate/FNR, and F1-Score are other parameters often used to evaluate the classification performance of neural networks. The precision and recall of the model should be as high as possible, whereas the FPR and FNR should be as low as possible. The F1-Score, which is the weighted average of precision and recall, was computed to evaluate whether the models were good when there was an uneven class distribution between drowsiness and alertness. Specifically, in our study, the dataset used suffers from a class imbalance; hence, the F1-Score provides useful insight into how good the different classifiers are in handling the imbalance. This measure can be used to evaluate whether a model performs well despite an imbalance in the dataset.

From the evaluation metrics (shown in Table 4) of all the trained models, the hyperparameter-tuned ensemble of the boosted tree classifier using AdaBoost had the lowest FPR and FNR, and hence, it is the best model based on the dataset used in this study. The ROC–AUC value also supports this statement, as the model has a higher separability value than the other models. However, the ensemble of bagged tree classifiers did not show much improvement after hyperparameter tuning, and one of the reasons might be the high number of splits, which was 302. Decision trees are the base learners for ensemble classifiers, and a higher number of splits in the decision tree makes the model more complex. The AdaBoost algorithm assigns equal weights to each sample of the training dataset when training the first weak learner. The subsequent weak learner model is trained using the recalculated weights of the sample to present a misclassification from the previous model. The algorithm makes predictions by recalculating the weighted average of the weak learners, which improves the predictive performance of the trained model.

The ensemble classifier is robust and gives the best performance among all supervised machine learning algorithms. The model uses multiple decision trees as base learners instead of considering only one. Taking the most common or average prediction for multiple decision trees renders the model more reliable than a single prediction model. Without hyperparameter tuning, the ensemble of bagged trees provided better performance. After hyperparameter tuning, the ensemble of boosted trees using AdaBoost provided the best performance among all the models used in this study. Bayesian optimization also helped improve the performance by keeping track of past evaluation results used to form a probabilistic representation of the model performance. It builds a probability model of the objective function and selects the most promising hyperparameters to evaluate the actual objective function. Of the many hyperparameters available, only the significant parameters are tuned to have the greatest effect on the ensemble classifier model result.

## 5. Conclusions

This study was performed on a public dataset of 12 subjects with EEG signals marked as alert or drowsy. Different ML models were trained to classify the observations into drowsiness and alertness, and their performances were evaluated based on different evaluation metrics. Previous studies have demonstrated that eyeblink-related parameters are good indicators of drowsiness. Therefore, in this study, eyeblink artifacts were used to detect drowsiness among drivers. Previous EEG-based driver drowsiness detection systems investigated brain rhymes such, as alpha, beta, and gamma to extract the features and train different models to predict drowsiness. The proposed work presents a novel way of detecting driver drowsiness using eyeblink artifacts extracted from EEG signals and the application of machine learning. The BLINKER algorithm was used to extract blink-related features from EEG signals. The observations obtained from BLINKER were cleaned and preprocessed before use for feature selection and model training. The medium-tree-based embedded feature selection technique selects the most useful features to improve the predictive performance of the ML classification models. For the classical ML models, we selected DT, KNN, SVM, and ensemble-bagged and boosted tree classifications. These models were further optimized using Bayesian optimization to obtain improved performance.

Among the classical ML models, the Bayesian-optimized AdaBoost classifier yielded the best performance, with an accuracy of 91.10%, TPR of 91.0%, FPR of 8.8%, FNR of 6.7%, precision of 88.2%, AUC of 0.97, and F1-Score of 0.9. The high F1-Score also indicates that the Bayesian-optimized AdaBoost classifier performs well with our dataset, which has class imbalance issues. The main contribution of this work is that we have shown that there is significant information that can be extracted from ocular or eyeblink artifacts present in EEG signals. While in most work involving EEG analysis, these artifacts have been dismissed as noise or unwanted signals, we have shown that for the right application, this “unwanted” signal may hold valuable information. This paper has shown that it is viable to use the features extracted from eyeblink artifacts in EEG signals for classifying drowsy and alert states, and these features may be able to supplement drowsiness detection techniques based on EEG signals. As opposed to driver drowsiness detection that is based on hybrid EEG–EOG information, our work suggests that we are able to also obtain ocular information without the placement of EOG sensors around the eye area.

One of the limitations of this study is the existence of class imbalance in the dataset. Therefore, the accuracy of the classification models should not be the only metric for evaluating the model performance. The F1-Scores for the best ML model in this study indicate that the models can handle class imbalance well. Another limitation of this study is that only one feature selection method was explored while investigating the classical ML models. Suggested future work includes solving the class imbalance problem using oversampling and undersampling, testing these models on different datasets, and using different feature selection techniques for the ML model.

## Figures and Tables

**Table 1:** Different methods of drowsiness detection.

Categories	Features	Measurements	Sensors
Vehicle-based	Vehicular	Steering wheel movement [4]	Attached to the vehicle
		Angular velocity [5]	
		Acceleration [4]	
		Lateral distance [6]	
Driver-based	Behavioral	PERCLOS [7]	Not attached to the driver
		PATECP [8]	
		PATMIO/yawning [9]	
		Blinking [10]	
		Gaze detection [11]	
		Head pose [11]	
		Facial expression [12]	
		Hand motion [13]	
	Physiological	EEG [14]	Attached to the driver
		ECG [15]	
		EOG [16]	
		EMG [17]	
		Skin responses (GSR & PPG) [18,19]	
		fNIRS [20]	

**Table 2:** Features from BLINKER and their description.

Feature Name	Feature Description
Duration Base (DB)	The difference between rightBase and leftBase to determine the blink length in seconds.
Duration Zero (DZ)	The difference between rightZero and leftZero to determine the blink length in seconds.
Duration Tent (DT)	The difference between rightZero and leftZero to determine the blink length in seconds.
Duration Half Base (DHB)	The difference between the frame defining the left-half base amplitude and the first intersection of the horizontal line drawn from the blink value at that point to the downstroke of the blink is the length of the blink in seconds.
Duration Half Zero (DHZ)	The difference between the frame indicating the left-half zero amplitude and the first intersection of the horizontal line drawn from the blink value at that point to the downstroke of the blink is the length of the blink in seconds.
Inter Blink Maximum Amplitude (IBMA)	Length of the intervals of any successive blink peaks in seconds.
Inter Blink Maximum Velocity Base (IBMVB)	The time in seconds between one blink’s maximum positive velocity (estimated from leftBase) and the following blink’s maximum positive velocity (calculated from leftBase).
Inter Blink Maximum Velocity Zero (IBMVZ)	The time in seconds between one blink’s maximum positive velocity (estimated from leftZero) and the following blink’s maximum positive velocity (calculated from leftZero).
Negative Amplitude Velocity Ratio Base (NAVRB)	The AVR (amplitude velocity ratio) computed using the maxBlink to rightBase interval.
Positive Amplitude Velocity Ratio Base (PAVRB)	The VAR computed using the leftBase to maxBlink interval.
Negative Amplitude Velocity Ratio Zero (NAVRZ)	The VAR computed using the maxBlink to rightZero interval.
Positive Amplitude Velocity Ratio Zero (PAVRZ)	The amplitude velocity ratio computed using the leftZero to maxBlink interval.
Negative Amplitude Velocity Ratio Tent (NAVRT)	The right tent line’s slope and the tent peak of any blink to compute the amplitude velocity ratio.
Positive Amplitude Velocity Ratio Tent (PAVRT)	The tent peak and slope of the left tent line to determine the amplitude velocity ratio.
Time Shut Base (TSB)	From the leftBase, the blink closest to 90% of its amplitude.
Time Shut Zero (TSZ)	From the leftZero, the blink closest to 90% of its amplitude.
Time Shut Tent (TST)	The blink closest to 90% of the tent peak height calculated in seconds.
Peak Max Blink (PMB)	The maximum amplitude of any blink.
Closing Time Zero (CTZ)	Difference between the maxFrame and leftZero calculated in seconds.
Reopening Time Zero (RTZ)	Difference between the rightZero and maxFrame calculated in seconds.
Closing Time Tent (CTT)	Difference calculated in seconds between the LeftxIntersect and xIntercept frames that create the tent.
Reopening Time Tent (RTT)	Difference calculated in seconds between the xIntersect and RightxIntercept frames that create the tent.
Peak Time Blink (PTB)	The maximum blink time in seconds since the beginning of the file.
Peak Time Tent (PTT)	Time in seconds since the beginning of the file of the tent’s peak.
Peak Max Blink (PMB)	Maximum blink amplitude.
Peak Max Tent (PMT)	Maximum tent peak height.

**Table 3:** The number of observations obtained from the BLINKER algorithm.

Subjects	Number of Samples Obtained from BLINKER	
	Alert	Drowsy
Subject 1	0	94
Subject 2	197	50
Subject 3	45	40
Subject 4	25	86
Subject 5	48	47
Subject 6	105	182
Subject 7	53	44
Subject 8	15	58
Subject 9	182	264
Subject 10	79	156
Subject 11	61	75
Subject 12	13	45

**Table 4:** Performances of the classification models.

Model	Performance Metrics	Before Hyperparameter Tuning	After Hyperparameter Tuning	Tuned Hyperparameters and the Optimal Values
Decision Tree	TPR (%)	77.60	76.60	Maximum number of splits: 211 Split criterion: Gini’s diversity index
	FPR (%)	18.00	16.80	
	FNR (%)	22.40	16.90	
	Precision (%)	75.80	76.70	
	Accuracy (%)	80.20	80.40	
	F1 score	0.77	0.77	
	AUC	0.82	0.82	
KNN	TPR (%)	82.10	86.50	Number of neighbors: 3 Distance metric: Mahalanobis Distance weight: Squared inverse
	FPR (%)	14.50	12.60	
	FNR (%)	17.90	10.10	
	Precision (%)	80.30	83.10	
	Accuracy (%)	84.10	87.00	
	F1 score	0.81	0.85	
	AUC	0.90	0.93	
SVM	TPR (%)	75.20	82.50	Box constraint level: 28.9228 Kernel scale: 1 Kernel function: Cubic
	FPR (%)	12.10	11.10	
	FNR (%)	16.90	12.50	
	Precision (%)	81.70	84.20	
	Accuracy (%)	82.50	86.20	
	F1 score	0.78	0.83	
	AUC	0.91	0.91	
Ensemble of Bagged Trees	TPR (%)	84.00	84.20	Number of learners: 100, Maximum number of splits: 84 Number-of-Predictors-to-Sample: 8.
	FPR (%)	14.10	12.20	
	FNR (%)	11.80	15.40	
	Precision (%)	81.00	83.20	
	Accuracy (%)	85.10	86.40	
	F1 score	0.83	0.84	
	AUC	0.93	0.94	
Ensemble of Boosted Trees (AdaBoost)	TPR (%)	79.70	91.00	
	FPR (%)	21.30	08.80	
	FNR (%)	15.70	06.70	
	Precision (%)	72.80	88.20	
	Accuracy (%)	79.10	91.10	
	F1 score	0.76	0.90	
	AUC	0.88	0.97	

## Data Availability

This study used a public EEG dataset of 12 subjects including EEG signals of drowsy and alert state. The original article where this dataset was obtained is reference [44] and the dataset is available at https://figshare.com/articles/dataset/The_original_EEG_data_for_driver_fatigue_detection/5202739 (accessed on 12 May 2022).
